# Influence of Polyethylene Glycol on Leaf Anatomy, Stomatal Behavior, Water Loss, and Some Physiological Traits of Date Palm Plantlets Grown *In Vitro* and *Ex Vitro*

**DOI:** 10.3390/plants9111440

**Published:** 2020-10-26

**Authors:** Amal F. M. Zein El Din, Mohamed F. M. Ibrahim, Reham Farag, Hany G. Abd El-Gawad, Ahmed El-Banhawy, Ibrahim A. Alaraidh, Younes M. Rashad, Islam Lashin, Ahmed Abou El-Yazied, Amr Elkelish, Ola H. Abd Elbar

**Affiliations:** 1 The Central Laboratory for Date Palm Researches and Development, Agricultural Research Center, Giza 12619, Egypt; amal.zeineldin@arc.sci.eg; 2 Department of Agricultural Botany, Faculty of Agriculture, Ain Shams University, Cairo 11566, Egypt; Reham_hassan@agr.asu.edu.eg; 3 Department of Horticulture, Faculty of Agriculture, Ain Shams University, Cairo 11566, Egypt; hany_gamal2005@agr.asu.edu.eg (H.G.A.E.-G.); ahmed_abdelhafez2@agr.asu.edu.eg (A.A.E.-Y.); 4 Botany Department, Faculty of Science, Suez Canal University, Ismailia 41522, Egypt; ahmedbanhawy@science.suez.edu.eg; 5 Department of Botany and Microbiology, College of Science, King Saud University, Riyadh 2455, Saudi Arabia; ialaraidh@ksu.edu.sa; 6 Plant Protection and Biomolecular Diagnosis Department, Arid Lands Cultivation Research Institute, City of Scientific Research and Technological Applications (SRTA-City), New Borg El-Arab City 21934, Egypt; Yrashad@srtacity.sci.eg; 7 Botany and Microbiology Department, Faculty of Science, Al-Azhar University, Nasr City 11651, Cairo, Egypt; islam79@azhar.edu.eg

**Keywords:** *Phoenix dactylifera*, *in vitro* hardening, polyethylene glycol (PEG), leaf anatomy, stomata, water loss %, *ex vitro* transfer, survival

## Abstract

Few reports explain the mechanism of PEG action on stomatal behavior and anatomical structure and analyze the photosynthetic pigments of *in vitro* date palm plantlets for better tolerance to *ex vitro* exposure. The main challenge for *in vitro* micropropagation of date palm techniques remains restricted to high survival rates and vigorous growth after *ex vitro* transplantation. *In vitro* hardening is induced by Polyethylene glycol PEG (0.0, 10, 20, 30 g L^−1^) for 4 weeks. Leaf anatomy, stomatal behavior, water loss %, photosynthetic pigments, and reducing sugars were examined in date palm plantlets (*Phoenix dactylifera* L.) cv. (Sewi) after 4 weeks from *in vitro* PEG treatment and after 4 weeks from *ex vitro* transplanting to the greenhouse. Leaf anatomy and the surface ultrastructure of *in vitro* untreated leaves showed a thin cuticle layer, wide opened malfunctioning stomata, and abnormal leaf anatomy. Furthermore, addition of PEG resulted in increasing cuticle thickness, epicuticular wax depositions, and plastids density, improving the stomatal ability to close and decreasing the stomatal aperture length while reducing the substomatal chambers and intercellular spaces in the mesophyll. As a result, a significant reduction in water loss % was observed in both *in vitro* and *ex vitro* PEG treated leaves as compared to untreated ones, which exhibited rapid wilting when exposed to low humidity for 4 h. PEG application significantly increased Chlorophylls a, b and carotenoids concentrations, especially 10, 20 g L^−1^ treatments, which were sequentially reflected in increasing the reducing sugar concentration. However, leaves of plantlets treated with PEG at 30 g L^−1^ became yellow and had necrosis ends with death. *In vitro* hardening by 20 g L^−1^ PEG increased the survival rate of plantlets to 90% after *ex vitro* transfer compared to 63% recorded for the untreated plantlets. Therefore, this application provides normal date palm plantlets developed faster and enhances survival after *ex vitro* transfer.

## 1. Introduction

Date palm (*Phoenix dactylifera* L.) is one of the most important fruit trees in arid and semiarid regions of the Middle East and North Africa [1]. Palm trees have been planted by separating the offshoots and independently established in the fields. However, this traditional method is not sufficient due to the limited number of offshoots produced from the mother tree in its lifespan. In addition, the seed propagated plants show great genetic variations and need up to seven years to reach the fruiting stage [2]. *In vitro* micropropagation techniques became effective methods for rapid and mass multiplication by either somatic embryogenesis [3,4] or organogenesis protocols [5,6]. However, the success of these protocols on a commercial scale is highly dependent on the ability of regenerated plantlets to transfer from *in vitro* controlled conditions and well established in the newly hard *ex vitro* conditions.

During *in vitro* culture, plantlets are exposed to special circumstances such as high relative humidity, low light intensity, and limited inflow of CO_2_. These special conditions lead to the formation of plantlets suffering from morphological and anatomical abnormalities which adversely influence water relations, photosynthesis efficiency, and other physiological processes [7]. These plantlets might easily be deteriorated by sudden changes in environmental conditions after their transfer to the greenhouse. Thus, plantlets need a period of acclimatization to eliminate these abnormalities, keep water balance, and consequently increase the survival rates [8,9,10,11].

The critical period for survival occurs within the first ten days to two weeks after transfer from *in vitro* to the greenhouse. During this period, plants are particularly fragile [12]. To increase survival rates during this period, plants from tissue culture are acclimatized by gradually decreasing relative humidity and increasing irradiance, CO_2_ concentration, or adding osmotic agents to reduce water availability of the culture medium [9,10,13,14].

Two principal problems facing date palm tissue culture technique are the low survival and slow growth rates of plantlets after they are moved from *in vitro* to *ex vitro* conditions during acclimatization [15]. A survival rate ranging from 40–50% has been reported for some date palm cultivars by Awad et al. [16] and Zaid and de Wet [17]. The low survival and slow growth rates might result from a lack of structural complexity of the epicuticular that distinguished the wide varieties of plants grown *in vitro* and their counterparts grown *ex vitro* [18,19,20]. In addition, high stomatal and cuticular transpiration rates of leaves increased water loss and desiccation when plantlets were taken out of the culture vessels to the greenhouse conditions [13,14,21,22,23].

Several reports have revealed polyethene glycol (PEG) as an inert osmotic agent to the culture medium during the rooting stage of plantlets to induce water stress before transferring to *ex vitro* [24,25]. PEG can reduce water availability in the *in vitro* culture medium *via* the decreasing water potential of nutrient solutions; thus, it can mimic drought stress which occurs in the greenhouse or in the open field [26,27]. PEG application is likely to repair the anatomical abnormalities by increasing epicuticular wax depositions [10] and causing stomatal closure [28,29], consequently reducing water loss. These effects may increase the plantlet ability to survive with a high rate. However, there are no sufficient reports that give details about the mechanism of stomatal closure or show how to eliminate the anatomical and physiological abnormalities in *in vitro* date palm leaves treated with PEG.

This study hypothesized that changing water availability of *in vitro* date palm plantlets by adding PEG to the culture media would generate functional stomata, modifying the anatomical and physiological abnormalities, and, consequently, would induce the development of more normal plantlets. This may be useful for a better understanding of the mechanisms that enable plantlets to overcome uncontrolled water loss, enhancing survival percentage after transferring *ex vitro*.

## 2. Results and Discussion

### 2.1. Anatomical Examinations

**Figure 1:**
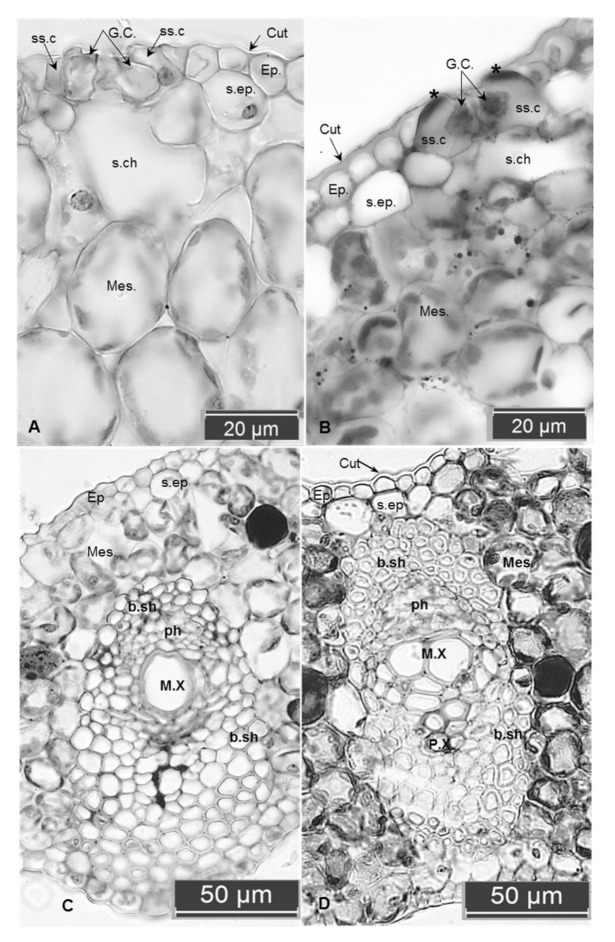
Cross-section of *in vitro* leaves of a date palm: (**A**,**C**) control untreated leaves, (**B**,**D**) treated with 20 g L^−1^ PEG. (**A**) The thin cuticle layer, larger mesophyll cells with fewer chloroplasts, greater substomatal chamber and intercellular spaces. Note the opened stomata and the same thickness of both ventral and dorsal walls of the guard cells, thin-walled of subsidiary cells. (**B**) The thick cuticle, compacted mesophyll cells with smaller substomatal chambers. Note the closed stomata, increasing in the thickness of ventral walls of the guard cell, and also the outer tangential walls of the two subsidiary cells appear lignified (the two stars). (**C**) Weak differentiation of the vascular bundle sheath compared to the well-developed one appears as lignified cells around phloem and xylem in (**D**). Abbreviations: Cut. cuticle; Ep. epidermis; GC. guard cells; ss.c. subsidiary cells; s. ep. sub epidermal layer; s.ch. substomatal chamber’ mes. mesophyll; b.sh. bundle sheath; ph. phloem; M.X. meta xylem; P.X. proto xylem.

Transverse sections of date palm leaves (control and PEG treated) under *in vitro* and *ex vitro* conditions showed differences in certain anatomical features. The *in vitro* control leaf exhibited the presence of a uniseriate epidermal layer on both adaxial and abaxial surfaces. It is covered with a thin layer of the cuticle (Figure 1A) compared with thick covering in the PEG treated leaves (Figure 1B). The lack or poor formation of the cuticle is common in plants grown under *in vitro* conditions [30], where high relative humidity inside the culture vessels represses cuticle formation [24]. This layer is an important factor for surviving the *in vitro* plantlets during transplantation to the *ex vitro* conditions, *via* helping in control transpiration passive water loss [31]. This may explain the increase in water loss rates in both *in vitro* and *ex vitro* untreated leaves compared to the PEG treated plantlets after 4 h of air drying treatment.

The *in vitro* control leaf is unifacial and amphistomatic. The stomata are tetracytic type. The guard cells are at the same level as the epidermal cells and have a wide opened elliptical shape. Observations indicated that both ventral and dorsal cell walls of the guard cells have the same thickness, this abnormal property causing open stomata and a failure to close (Figure 1A); whereas the stomata in the PEG-treated leaves have additional thickening occurred in the ventral walls of the guard cells (Figure 1B). Variations in the thickness of ventral and dorsal cell walls of the guard cells appear to be involved with the mechanism of stomatal movements. This result can be explained by Evert [32], who pointed out that the guard cells of the major taxa have their distinguishing characteristics; however, all share a notable feature that is characterized by the presence of unevenly thickened walls. This feature appears to be related to the changes in shape and volume (and the associated changes in the stomatal aperture size) caused by changes in turgor pressure within the guard cells. In addition, an obvious increment in thickness and lignification was recorded in the outer tangential walls of the subsidiary cells (Figure 1B). This may contribute to controlling stomatal closure and acquiring some mechanical strength for the epidermis against water stress when the plantlets are exposed to the *ex vitro* conditions.

**Figure 2:**
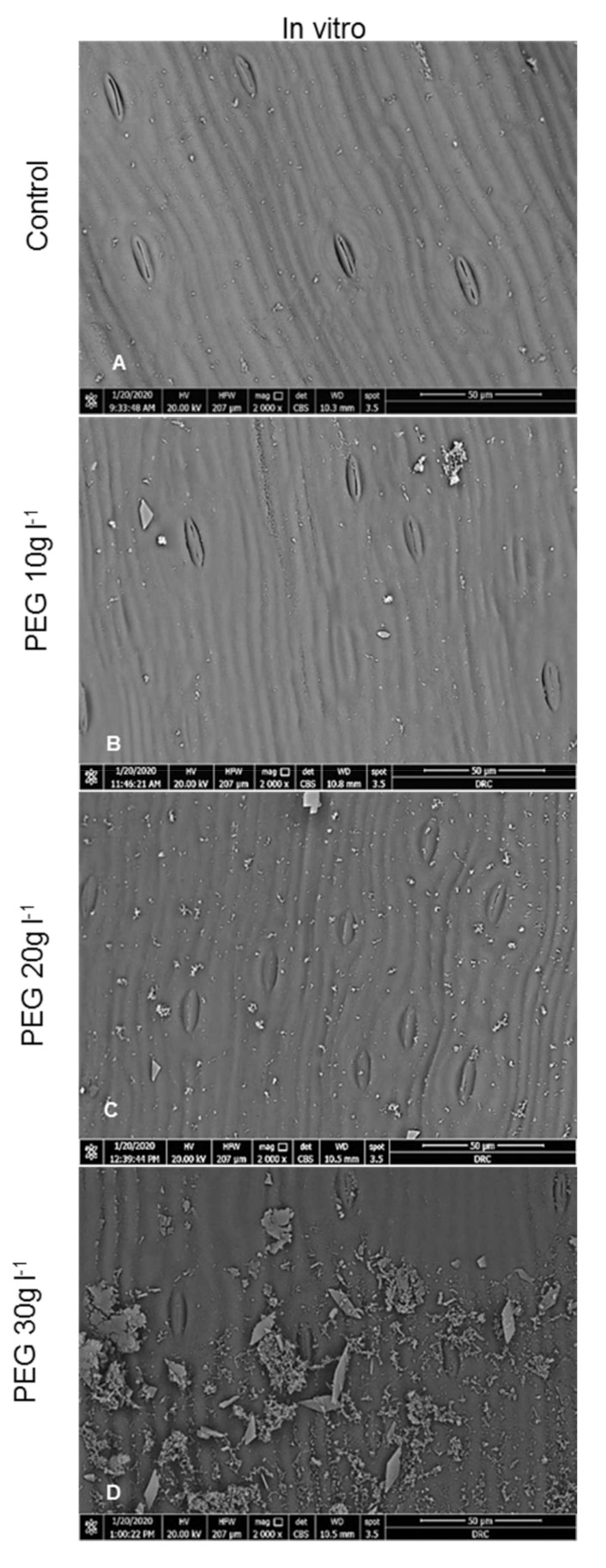

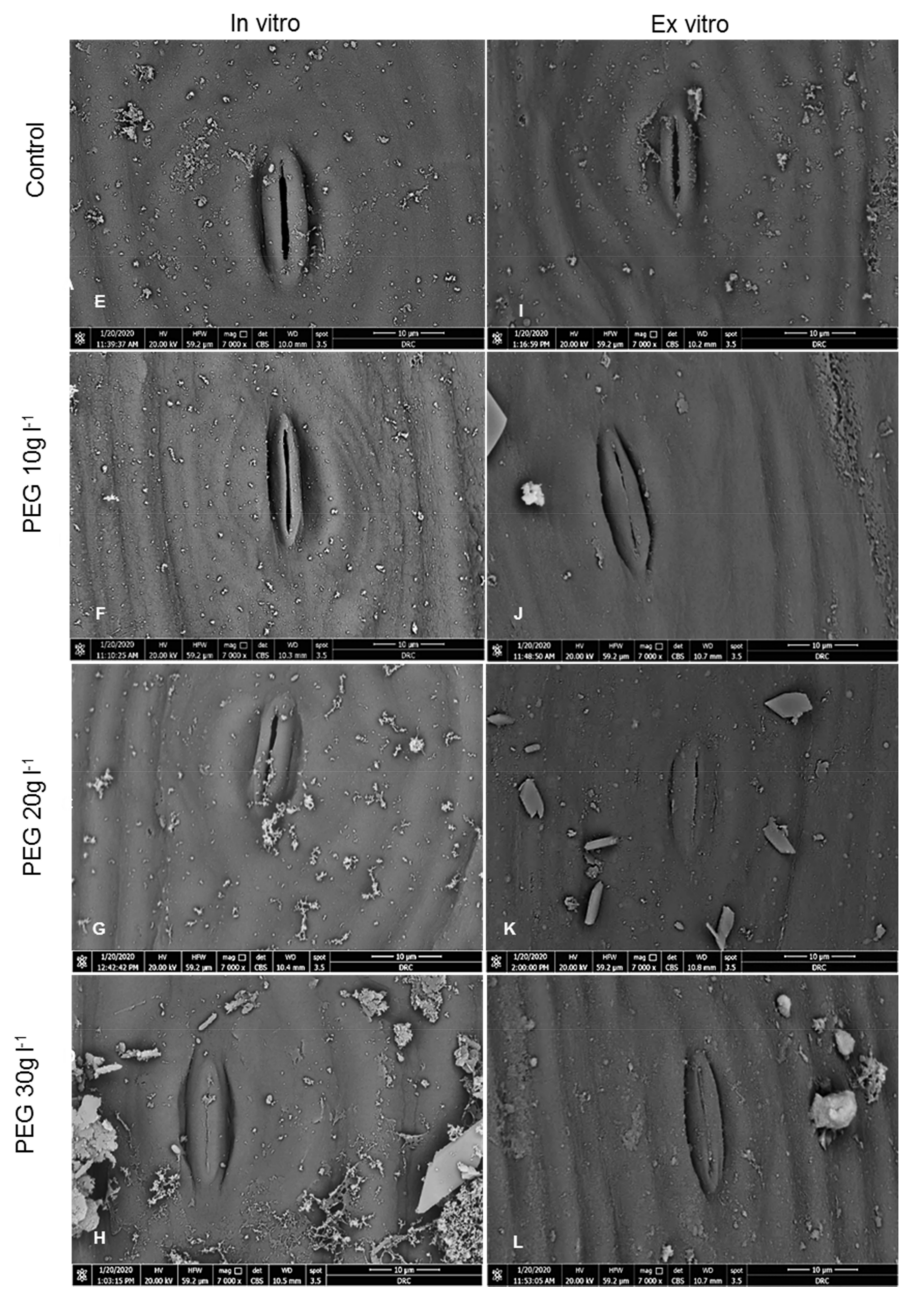
SEM micrographs of the adaxial surface of *in vitro* date palm (cv. Sewi) grown in media supplemented with different levels of PEG. (**A**) shows that most stomata are wide opened, the leaf surface apparently smooth. (**B**) shows fewer opened with narrow apertures, the development of little epicuticular wax on the leaf surface. (**C**) shows increasing closed stomata, as well as the density of the epicuticular wax density compared to (**A**,**D**). Most stomata are closed covered with a high density of epicuticular wax crystalloids. (Magnification: 2000×, Scale Bar = 50 µm). (**E**–**H**) are the magnified micrographs from (**A**–**D**) respectively. (**E**) Guard cells are full, slightly raised, stomatal aperture is wide compared to narrow ones observed in (**F**,**G**). (**H**) shows a completely closed stomata with an increase in epicuticular wax density on the leaf surface. (**I**–**L**) are the adaxial surfaces of leaves after four weeks from *ex vitro* transplantation. (**I**) shows stomata still opened, slightly raised up from the leaf surface. (**J**,**K**) shows a decrease in stomatal apertures, guard cells are slightly depressed. (**L**.) showed a completely closed stomata with increase in epicuticular wax density. (Magnification: 7000×, Scale Bar = 10 µm).

The SEM observations showed that no significant differences were observed in the number of stomata between treatments, whereas the number of opened stomata and the stomatal aperture length were significantly different (Table 1 and Table 2). The high frequency of opened stomata with large stomatal apertures were recorded in the *in vitro* control leaves; by contrast, the PEG treated leaves under *in vitro* and *ex vitro* conditions had fewer opened stomata with the narrowest apertures (Figure 2 and Table 1 and Table 2). This result is in agreement with several reports in which large stomatal apertures were recorded in the *in vitro* leaves when compared with the same leaves grown *ex vitro* [10,29]. The stomata of the *in vitro* leaves initiated and developed under high relative humidity conditions having malfunctioning disability to close [10,29,33,34]. When these *in vitro* plantlets transfer to the *ex vitro*, the stomata remain open resulting in rapidly increasing water loss and causing wilting due to excessive transpiration through the large apertures of these nonfunctioning stomata [35,36]. PEG can stimulate the effect of drought stress in culture media. Thus, stomatal closure is the most effective mechanism to combat water stress *via* increasing the thickness and lignification of the ventral walls of the guard cells over the dorsal ones. It is well documented that water stress is responsible for increase in cell wall lignification [37,38]. The size of stomatal aperture is regulated mainly by the turgor pressure of the guard cell which was decreased under drought stress [39]. This mechanism was linked with an increase in the levels of endogenous abscisic acid (ABA) in leaves under drought stress [40]. Results indicated that PEG as an osmotic agent was able to recover the function of the stomata and reduce water loss % then enhance the survival rate (as shown below) after transfer to the *ex vitro* conditions. Confirmatory observations on PEG treatments during the rooting stage were made to reduce the water loss of the date palm [20,25].

With a high magnification of SEM observations, PEG-treated plants under *in vitro* and *ex vitro* conditions exhibited well-defined epicuticular wax forming crystalline deposits scattered on leaf surfaces. These epicuticular wax crystals were very rare and less dense on the *in vitro* untreated leaves as compared to the PEG treated leaves under *in vitro* or the *ex vitro* conditions (Figure 2). The reduction in epicuticular wax density may cause an increase in the cuticular conductance of water vapor and contribute to water loss rapidly (Table 3). The *in vitro* hardening of PEG reduced stomatal apertures, recovered stomatal functioning, stimulated a more thick cuticle layer and formed a high-density epicuticular wax deposition (Figure 2). Increasing cuticle thickness was a prevalent mechanism against drought stress *via* protecting cells from dehydration, consequently reducing leaf cuticular permeability and reducing water loss [41,42]. Additionally, the epicuticular wax crystalloids could protect the leaf surface from drought by enhancing the solar radiation reflection and reducing leaf temperature and transpiration rates [43]. Thereby, these mechanisms contribute to wilting avoidance after transplant to the *ex vitro* conditions.

Despite this, PEG has been used as an osmotic agent by imposing water stress on plants and it has been widely used to induce the formation of epicuticular wax grown under *in vitro* conditions [28,44]. However, the result indicated that treatment with PEG 30 g L^−1^ caused closing for most stomata and in many cases the apertures covered by epicuticular wax deposits. This result has negative effects and causes death for the external leaves. One possible explanation for this finding is reported by Arve et al. [40]. They found that on reduction of stomatal aperture, little CO_2_ is uptaken and consequently the transpiration rate is lowered. By controlling the size of stomatal apertures, plants can regulate the amount of water loss even with a sacrifice of CO_2_ uptake during unfavorable conditions; but continued stomatal closure means low CO_2_ capture, less water and nutrient uptake, with consequently negative effects on photosynthesis process.

Beneath the outer and inner epidermises, hypodermis developed as a continuous colorless layer, increased into two to three layers in the major veins zones. This layer acquired thicker walls in the PEG-treated leaves (Figure 1A,B). This may contribute against water loss through leaf surfaces during Limited moisture levels.

**Figure 3:**
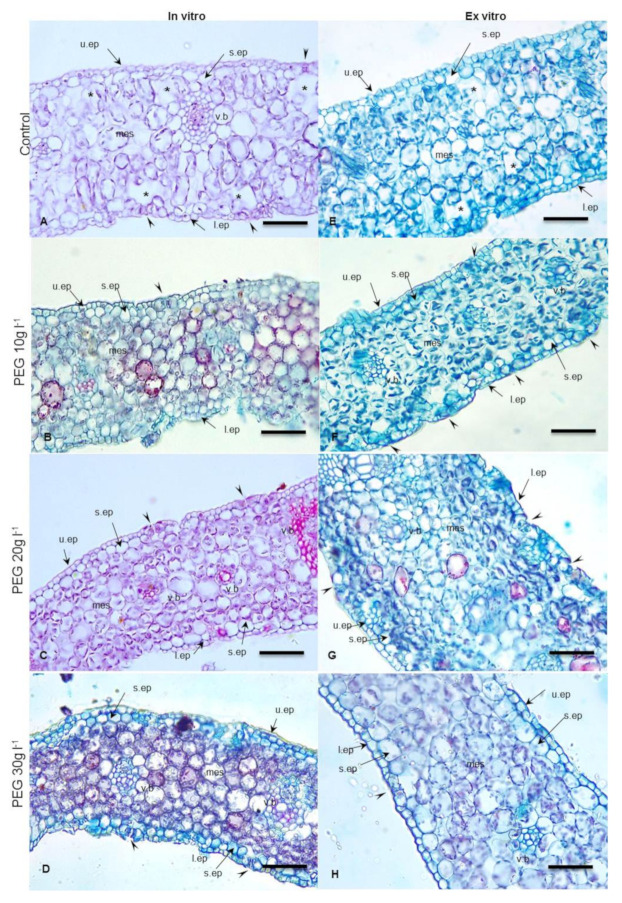
Cross sections in *in vitro* date palm leaves (cv. Sewi) grown in media supplemented with different levels of PEG (**A**–**D**) and after 4 weeks from *ex vitro* transplantation (**E**–**H**). (**A**) Abnormal structures appeared as thin cuticle, large mesophyll cells and fewer chloroplasts, large intercellular spaces, and substomatal chambers. (**B**,**C**) Organized mesophyll with reduction in intercellular spaces and well developed cuticle layer. (**D**) Compacted mesophyll, thick cuticle. (**E**) Leaves still have thin cuticle, loose mesophyll, but the plastids appear in higher density than observed in (**A**,**F**). More organized mesophyll, closed stomata. (**G**) Well developed compacted mesophyll and slightly sunken stomata. (**H**) Thick cuticle, closed stomata. Abbreviations: u.ep. upper epidermis; s.ep. subepdermal layer; l.ep. lower epidermis; mes. Mesophyll; v.b. vascular bundle. Arrowheads point to stomata, stars point to substomatal chambers and intercellular spaces. Bar = 200 µm.

The mesophyll of *in vitro* leaves is undifferentiated, consisting of 8–10 layers of chlorenchyma with noticeable intercellular spaces, large substomatal chambers, and poor mesophyll differentiation (Figure 3A). These features cause the *in vitro* leaves to be highly sensitive to transplantation shock (Figure 3E). However, the PEG-treated *in vitro* leaves (Figure 3B–D) and *ex vitro* ones (Figure 3F–H) have more organized and compacted mesophyll layers with less intercellular spaces without increasing the number of mesophyll layers. This result may be explained by the osmotic stress caused by PEG which can increase cell size [45]. Additionally, the result indicated that these compacted mesophyll cells have dense plastids and chlorophyll contents. By replacing the loose abnormal mesophyll layers with compacted ones, the leaves are safe from desiccation effects, since the extensive water loss recorded in the control *in vitro* and *ex vitro* leaves may be attributed to the large intercellular spaces between the loose mesophyll. In addition, the high density of plastids and chlorophyll contents may contribute to enhancing the photosynthetic process. Thus, it could be suggested that PEG improves the quality of the hardened plantlets *via* modifying stomatal functionality, controlling water loss, and increasing photosynthetic pigments content over the other untreated control plantlets.

The large veins in *in vitro* leaves are composed of vascular bundles surrounded by a continuous sheath. The minor veins are embedded in the mesophyll layers and consist of a few xylem and phloem elements (Figure 1C). This weak vasculature causes fragile leaves easily desiccated when exposed to *ex vitro* conditions; whereas leaves treated with PEG have well defined major and minor veins surrounded by lignified fibrous bundle sheath (Figure 1D). This lignified bundle sheath is likely to provide mechanical support to the cell wall of phloem and xylem elements against turgor pressure which is nearly decreased under water stress. This observation was in congruence with those obtained by Abd El-Maboud and Abd Elbar [46] and Abd Elbar et al. [47] through increasing lignification in different leaf tissues growing under drought and/or salinity stress. Lignification provides rigidity in the leaves, and when increasing in cells around the phloem indicates enhanced phloem resistance against obliteration caused by stress conditions. Additionally, increasing lignification in the bundle sheath or fibers around xylem elements helps in protecting water columns from embolism [47].

### 2.2. Water Loss %

**Figure 4:**
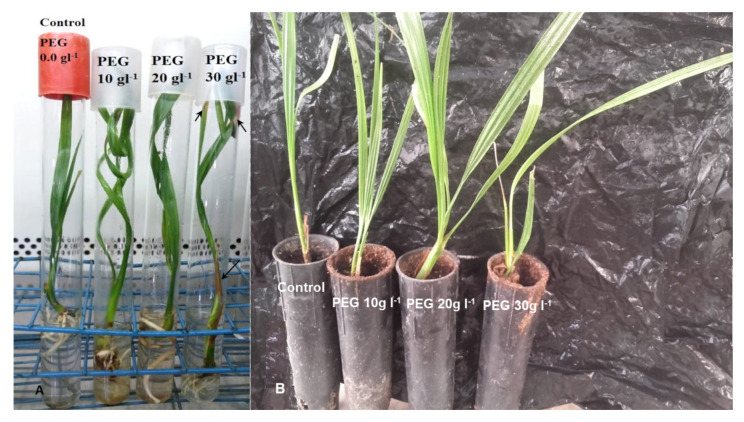
*In vitro* PEG hardening of date palm plantlets Sewi cv. (**A**) 4 weeks after *in vitro* treatments. Arrows point to browning regions in the external leaves treated with 30 g L^−1^ PEG. (**B**) Plantlets transfer to *ex vitro* conditions for another 4 weeks. Note: The 20 g L^−1^ PEG hardened plantlets appeared stronger and grew faster, and this enabled them to tolerate the low relative humidity in the greenhouse.

The data in Table 3 indicate that all treated and untreated *in vitro* and *ex vitro* detached leaves were subjected to the air-drying process showing an increase in water loss % by increasing time of drying. The *in vitro* hardening of PEG caused reduction in water loss % in both *in vitro* and *ex vitro* detached leaves. Although the treatment with 30 g L^−1^ PEG recorded the lowest reduction in water loss percent (38.42%), it caused a harmful effect such as the browning of the external leaves of *in vitro* hardened plantlets, and these leaves then died (Figure 4A).

The *in vitro* and *ex vitro* untreated leaves exhibited highly significant percentages of water loss (46.13 & 41.24, respectively). This can be explained by the occurrence of high relative humidity within the culture vessels in *in vitro* environment, which leads to the formation of abnormal plantlets which lack the mechanisms to control the excessive water loss during acclimatization.

The abnormal features observed in the *in vitro* control date palm leaves, such as the thin cuticle, malfunctioning stomata, large mesophyll cells and fewer chloroplasts, large intercellular spaces and substomatal chambers, were replaced with thick cuticle, functioning stomata, and compacted mesophyll in PEG treatments. Additionally, the numbers of closed and opened ones differ totally from PEG-treated and untreated plantlets. There was a gradual increase in the number of the closed stomata per mm^−2^ in both adaxial and abaxial surfaces by increasing the PEG concentration from 10 to 30 g L^−1^ while the number of in control plantlets was significantly low (Table 1 and Table 2). These data may reflect the importance of *in vitro* hardening treatment for inducing such modifications in leaf anatomy and stomatal behavior to reduce water loss from the foliar part. Hence, this will increase the survival rate of plantlets. Data obtained during this investigation accord with the previous data obtained by Pospíšilová [18]; Sutter [19]; Dami and Hughes [24].

The results indicated that the percentage of water loss in the *in vitro* control detached leaves was higher than that recorded in the *ex vitro* control ones (46.13 and 41.24%, respectively, Table 3). It is well known that plantlets grown *in vitro* might be easily wilted and damaged by a sudden transfer to the *ex vitro* conditions. Thus, these plantlets need several weeks under a shade and subjected to a decrease in the relative air humidity gradually to correct all abnormalities in their anatomy and physiology that were induced by special conditions of *in vitro* culture [9,13]. This is what actually followed for the date palm plantlets during *ex vitro* transfer for four weeks, as a result causing reduction in the water loss %.

### 2.3. Photosynthetic Pigments and Reducing Sugars

The Data in Table 4 indicate that the highest concentration of PEG (30 g L^−1^) caused a significant decrease of Chlorophyll a, Chlorophyll b, Chlorophyll a,b, and carotenoid concentrations. This result may be explained by the water stress induced by PEG leading to a reduction in photosynthetic traits such as chlorophyll concentration resulting in browning and the death of the external leaves. These results are in agreement with Cha-um et al. [48], who observed that osmotic stress induced by PEG caused degradation of Chl a, Chl b, and carotenoids in the leaves of oil palm seedlings. The authors observed that the leaves became chlorosis and then turned burned especially in severe water loss. On the other hand, the *in vitro* and *ex vitro* untreated plantlets recorded a significant decrease in Chl a, Chl b, Chl a,b, and carotenoid concentrations. This was attributed to the abnormal leaf anatomy and malformed stomata, although the plantlets appeared healthy, but were unable to be actively photosynthesizing. Hazarika et al. [7] explained that the *in vitro* plantlets characterized with the heterotrophic mode, poor in chlorophyll contents, were inactive in the enzymes required for photosynthesis even after transfer *ex vitro* because the photosynthetic system did not develop further.

Chlorophyll a,b, Chlorophyll a,b, and carotenoid concentrations exhibited a significant increase with 10, 20 g L^−1^ PEG *in vitro* hardening treatments, and the highest significant values (33.795, 11.267, 45.062, and 8.591 µg ml^−1^, respectively) were recorded with 20 g L^−1^ PEG. It is known that environmental stresses especially water stress and salinity increase the endogenous ABA concentration. Thus, ABA is considered to function as a stress hormone [49]. ABA treatment led to a reduction in the water loss of plantlets during transfer to *ex vitro* conditions [28,29]. Kong and Yeung [50] proved that PEG only had a slight effect on endogenous ABA levels in angiosperm and conifers. Thus, PEG may act as an anti-transpirant during hardening treatment by increasing the endogenous ABA levels in addition to its osmotic agent properties. Data obtained here indicated that the photosynthetic pigments content increased when plantlets were transplanted *ex vitro* in comparison with those which cultured *in vitro* in all PEG-treated and untreated plantlets. These results ensure the previous data obtained by Pospíšilová et al. [18] who reported that ABA treated *Nicotiana tabacum* plantlets had a high photosynthetic rate and the contents of photosynthetic pigments increased after two weeks from their transferred to *ex vitro* conditions. Additionally, the authors found that using anti-transpirants such as abscisic acid triggered the hardening of *in vitro* plantlets.

Regarding the reducing sugars, the data in Table 4 indicated that the highest significant content of reducing sugars (260.059 mg g^−1^) was recorded in *in vitro* hardened plantlets treated with 30 g L^−1^ PEG, whereas the lowest content was recorded in *in vitro* control plantlets (63.623 mg g^−1^). There was an increment in reducing sugars concentration by increasing PEG concentration. Generally, it is well established that the accumulation of organic solutes (osmolytes) such as reducing sugars is essential as a response for plants to avoid the adverse effect of water stress [51,52]. This explains the rise in the concentration of reducing sugars. Stasolla et al. [53] explained the reason for the rise of reducing sugars after PEG treatments by observing a high expression of some genes involved in sucrose catabolism and nitrogen assimilation in *in vitro* pine embryos. Additionally, Handa et al. [54] found that reducing sugars increased with decreasing the external water potential. They detected that sucrose within the cells was 3–8 fold lower than reducing sugars. Moreover, Zanella et al. [55] reported that using PEG as water stress increased endogenous methane (CH_4_) and sugar contents in maize. The authors suggested that sugars may act as osmoprotectants during *ex vitro* conditions. Thus, accumulation of reducing sugars was an important physiological adaptation against the new circumstances of water loss, *via* contributing the osmotic adjustment for the plant cell and maintaining their growth. Moreover, plantlets may restore these accumulated sugars in their persistent leaves as a source of energy, particularly in the first few days after transplanting *ex vitro*.

The transfer of hardened plantlets from *in vitro* to *ex vitro* conditions (for another four weeks) resulted in decreasing the concentration of the reducing sugar when compared to *in vitro* hardened plantlets and control except for the 30 g L^−1^ treatment. Noticeable well-growth features of hardened plantlets at 10 and 20 g L^−1^ PEG include higher growth rates, less physiological disorders, dark green color, and having stronger shoot and root systems (unpublished data) and a higher survival of plantlets. This result may be explained by the fact that an increase in reducing sugars is accompanied by an increase in the concentration of photosynthetic pigments. It is well known that reducing sugars are important for the differentiation process due to the necessity for the formation of reserves and cell wall polysaccharides [56].

Culturing the plantlets in *in vitro* closed vessels to prevent the microbial contamination limits the inflow of CO_2_ and outflow of gas. Additionally, lowering the light intensity and adding culture media with the sugars resulted in the formation of abnormal plantlets which suffered from some anatomical and physiological problems.

Obtained data in Table 4 revealed that control *in vitro* plantlets recorded the lowest content of reducing sugars concentration (63.623 mg g^−1^ DW). This could be related to the abnormal anatomical observations obtained during this investigation. Although the plantlets may appear “fully functional” physiologically, they are lazy or unlikely to be actively photosynthesizing—simply because it is unnecessary under *in vitro* conditions. Even if chlorophyll is present in the leaves, it is probable that the enzymes responsible for photosynthesis are inactive or absent, in addition to the abnormal leaf anatomy and malfunctioning stomata. Thus, the *in vitro* hardening by PEG may accelerate the development of such processes and reduce the time consumed in acclimation in the greenhouse.

### 2.4. Survival Percentage (%)

The obtained data in Table 5 and Figure 4 showed that the plantlets which hardened previously with 20 g L^−1^ PEG in the *in vitro* culture medium recorded the highest survival percentage (90%) followed by plantlets that hardened by adding 10 g L^−1^ PEG (72%). From the obtained data, it could be noticed that the photosynthetic pigments increased when the plantlets were moved to *ex vitro* conditions. This may be attributed to the high light intensity in the greenhouse in comparison with the tissue culture lab.

The success of the tissue culture technique for the commercial level depends on the ability of regenerated healthy plantlets to transfer out to the greenhouse with the high survival and faster growth rates [15,57]. Losing a large number of plantlets during *ex vitro* transferring mean losing the handling time consumed for the acclimatization period, especially for date palm. Date palm trees have a prolonged acclimatization period, taking from 15–24 months before transfer to an open field [15]. The damage or loss of the transferring plantlets is due to the unbalanced water relations [58]. Abnormal stomata lead to a failure in the control of the water loss by a transpiration process finally leading to wilting of the plantlets due to the low relative humidity within the greenhouse [18,19,59,60]. Thus, several studies aim to control the water loss by inducing water stress to modify the water availability for the growing plantlets [20,24,25].

Hardening of date palm plantlets in *in vitro* culture leads to a speedy *ex vitro* acclimatization process by decreasing the water loss rate. This period is highly important for plantlets to correct their anatomical and physiological abnormalities.

Pospisilova et al. [10] reported that plantlets need a period of time (several weeks) to correct their anatomical and physiological abnormal features induced by special environmental conditions in *in vitro* growth. This period is very important to increase the survival rate and ensure the success of plant growth in the greenhouse. Increasing the survival rate occurs by affecting the water loss rate and photosynthetic activities and products.

## 3. Materials and Methods

### 3.1. Plant Material and Growth Conditions

Date palm plantlets were derived *via* indirect somatic embryogenesis of Sewi cv. and thus used as explant material for this investigation. The study was performed in the tissue culture lab of the Central Lab for Date Palm Researches and Development, ARC, Egypt, and the Agricultural Botany Department, Faculty of Agriculture, Ain Shams University, during the period 2019–2020.

Individually rooted plantlets were selected, approximately uniform in size, number of leaves, and roots (2–3 leaves and 3–4 roots). Plantlets were cultured on ^1^/_4_ strength of MS [61] basal liquid media supplemented with 0.1 mg L-1 NAA plus 10 g L^−1^ sucrose and 0.2 g L^−1^ AC (activated charcoal). Polyethene glycol (PEG, molecular weight 8000, HIMEDIA REF RM 7402–500) was added at different concentrations (0, 10, 20, and 30 g L^−1^). The cultures were incubated under the recommended physical environmental conditions for date palms plantlets 27 ± 2 °C and 16 h illumination. The photosynthetic photon flux density (PPFD) was set at 40 µ mol m^−2^ s^−1^ using white, fluorescent lamps. Plantlets were transferred to the greenhouse after four weeks and incubated under the plastic tents for another four weeks. The relative humidity was decreased gradually to avoid the excessive desiccation resulting from their transfer from *in vitro* to *ex vitro* conditions [23]. All plantlets were fertilized by half strength of MS inorganic salts every 10 days. PEG treated and control plantlets grown in the greenhouse were rated for survival percentage.

### 3.2. Leaf Anatomy and Surface Ultrastructure (Scanning Electron Microscope, SEM)

For anatomical observations, samples were taken after 30 days for both *in vitro* and *ex vitro* PEG treatments. Small pieces were cut from the mid-portion of the lamina, fixed in FAA solution (Formalin, Acetic acid, and 50% ethyl alcohol 5:5:90) for 24 h. Progressive dehydration using the ascending concentration of ethanol was performed, and then embedding in Paraplast (Sigma Aldrich, St. Louis, MA, Paraplast plus p. 3688) according to the method described by Abdelbar [62]. Transverse sections 10 µm were made using the LEICA rotary microtome model RM 2125 RTS. Anatomical examinations were achieved using a LEICA light microscope model DM-500 supplemented with a digital camera LEICA ICC 50 HD with LAS E7 software version 2.1.0 2012.

For stomatal densities (number. mm^−2^) as well as stomatal aperture length (µm) and leaf surface ultrastructure, fresh leaf samples were excised and fixed in 3% glutaraldehyde for 24 h at 4 °C [63]. The specimens were dehydrated using acetone and coated with gold. The morphological examinations were achieved by a Quanta Scanning Electron Microscope (Quanta FEG 250). The photo-analysis was achieved using ImageJ software, and features were calculated as the average of 30 measurements.

### 3.3. Determination of the Water Loss %

The detached leaves obtained from the PEG treated and control plantlets (grown *in vitro* for four weeks and after being transplanted in the greenhouse for another four weeks) were subjected to air-drying treatment in the laminar cabinet (at 27 ± 2 °C). Then, the leaves were weighed immediately after detaching from intact plantlets and every 30 min for a 4 h period. Thereafter, they were dried in the oven for 24 h at 70 °C and reweighted to determine their dry weight. The percentage of water loss was calculated according to Dami and Hughes [24] with the subsequent formula:

WL = (FW_t0_ − DW_t_) − (FW_t_ − DW_t_) × 100/(FW_t0_ − DW_t_)

where WL = percent of water loss, FW_t0_ = fresh weight at zero-time, DW_t_ = dry weight, and FW_t_ = fresh weight after time.

### 3.4. Photosynthetic Pigments

Fresh leaves (0.3 g) were extracted by 10 mL (DMF) N, N dimethyl formamide [64]. Chlorophylls a, b and carotenoids were determined according to the method of [65]. Absorbance was measured in a UV/VIS spectrophotometer. The concentrations were calculated using the following equations:

Chl a (µg mL^−1^) = 12 A663.8 − 3.11 A646.8

Chl b (µg mL^−1^) = 20.78 A646.8 − 4.88 A663.8

Carotenoids (µg mL^−1^) = (1000 A470 − (1.12 Chl a − 34.07 Chl b))/245

### 3.5. Determination of Reducing Sugars

Fresh leaves (0.3 g) was extracted by ethanol 70%. The supernatant was evaporated to dryness, and then dissolved in 10 mL isopropanol 10%. Reducing sugars were determined as glucose (mg/100 g f. wt.) using 3,5-Dinitrosalicylic acid (DNS) at 540 nm according to Marsden et al. [66].

### 3.6. Statistical Analysis

The used design was a complete randomized design with 10 replicates, using least significant difference (LSD) test at 5% according to Snedecor and Cochran [67] and Gomez and Gomez [68].

## 4. Conclusions

The vigor of regenerated plantlets to transplant successfully *ex vitro* is determined by their ability to overcome the water limitation in the greenhouse. Thus, a better understanding of the physiological and anatomical events occurring during the *in vitro* hardening process is very important to understand how the plantlets deal with such problems. In this study, using PEG as an osmotic agent resulting in a decrease of the water potential of hardening culture media, achieved the generation of more functional stomata, eliminated anatomical abnormalities, decreased water loss, increased photosynthetic pigments content, and finally increased the survival percentage of PEG-hardened plantlets after transfer to *ex vitro* conditions. Adding PEG 10, 20 g L^−1^ provides an efficient hardening protocol of date palm plantlets *via* controlling water loss and shortens the time required for acclimatization with greenhouse conditions.

## Figures and Tables

**Table 1:** Stomatal density (number mm^−2^), number of opened, closed stomata (number mm^−2^), and stomatal aperture length (μm) on adaxial and abaxial surfaces of date palm leaves grown *in vitro* after four weeks of PEG hardening treatment.

PEG g L^−1^	Adaxial Surface				Abaxial Surface			
	Density	Opened	Closed	Aperture Length	Density	Opened	Closed	Aperture Length
**0.0**	140.6	133.74 a	6.82 d	13.67 a	159.5	150.9 a	8.54 c	14.67 a
**10**	140.6	101.2 b	39.40 c	10.74 ab	154.4	114.9 b	39.42 b	11.32 b
**20**	138.9	75.46 c	63.42 b	9.54 bc	145.8	99.46 c	46.26 b	10.45 b
**30**	149.4	41.10 d	108.1 a	7.36 c	151.4	51.42 d	89.18 a	7.77 c
**LSD_0.05_**	NS	15.29	20.93	3.30	NS	14.78	16.46	1.305

Means followed by different letters are significantly different at *p* ≤ 0.05 according to least significant difference (LSD) test.

**Table 2:** Stomatal density (number mm^−2^), number of opened, closed stomata (number mm^−2^), and stomatal aperture length (μm) on adaxial and abaxial surfaces of date palm leaves four weeks after transplanting *ex vitro*.

PEG g L^−1^	Adaxial Surface				Abaxial Surface			
	Density	Opened	Closed	Aperture Length	Density	Opened	Closed	Aperture Length
**0.0**	145.8	109.7 a	36.04 c	11.44 a	187.0 a	145.8 a	41.16 b	12.32 a
**10**	147.7	86.02 b	61.66 b	7.87 b	158.0 b	101.6 b	56.44 b	8.71 b
**20**	154.3	41.12 c	115.0 a	6.15 c	168.1 ab	54.86 c	113.2 a	6.99 c
**30**	154.1	30.58 c	123.5 a	3.14 d	157.34 b	33.46 d	124.0 a	3.43 d
**LSD_0.05_**	NS	14.06	20.15	1.59	19.54	17.75	21.28	1.181

Means followed by different letters are significantly different at *p* ≤ 0.05 according to LSD test.

**Table 3:** Effect of PEG applications on water loss % of the *in vitro* and *ex vitro* detached leaves of date palm plantlets during 4 h air drying treatments.

Time (A)	PEG g L^−1^ (B)				Mean (A)
	0.0	10	20	30	
***In vitro***					
**30 min**	24.19 q	22.94 q	22.91 q	20.43 r	20.62 H
**60 min**	36.30 n	33.35 o	33.38 o	30.33 *p*	33.27 G
**90 min**	42.24 jk	38.76 m	36.61 n	34.92 no	38.13 F
**120 min**	44.52 i	43.93 ij	39.64 lm	38.74 m	41.71 E
**150 min**	50.54 de	46.85 h	42.57 jk	41.30 kl	45.32 D
**180 min**	54.46 c	51.67 d	46.55 h	44.64 i	49.33 C
**210 min**	56.67 b	53.83 c	48.68 fg	47.81 gh	51.75 B
**240 min**	60.38 a	55.31 bc	50.28 Ef	49.22 efg	53.80 A
**Mean (B)**	46.13 A	43.33 B	40.03 C	38.42 D	
***Ex vitro***					
**30 min**	22.32 n	21.19 no	20.43 o	18.35 p	20.6 H
**60 min**	33.13 k	31.11 l	30.34 l	24.49 m	29.77 G
**90 min**	36.63 hi	34.50 jk	33.35 k	30.85 l	33.83 F
**120 min**	39.50 g	37.30 h	35.30 ij	33.27 k	36.35 E
**150 min**	42.93 ef	40.28 g	37.78 h	36.37 hi	39.34 D
**180 min**	48.18 cd	44.54 e	40.81 g	40.36 g	43.47 C
**210 min**	51.57 b	46.93 d	42.82 f	42.80 f	46.03 B
**240 min**	55.66 a	49.33 c	46.60 d	43.97 ef	48.89 A
**Mean (B)**	41.24 A	38.16 B	35.93 C	33.81 D	
**LSD_0.05_ for**		***In vitro***		***Ex vitro***	
**A**		**0.4913**		**0.5907**	
**B**		**0.6949**		**0.8354**	
**AB**		**1.702**		**1.671**	

Means followed by different letters are significantly different at *p* ≤ 0.05 according to LSD test. (A) Mean of water loss after time (30 min intervals). (B) Mean of PEG concentrations.

**Table 4:** Effect of PEG application on Chl a, Chl b, and Chl a, b, carotenoids and reducing sugars concentrations in leaves of date palm plantlets after 4 weeks from *in vitro* PEG treatments and 4 weeks from *ex vitro* transfer.

PEG g L^−1^	Chl_a_ µg mL^−1^	Chl_b_ µg mL^−1^	Chl_a,b_ µg ml^−1^	Carotenoids µg mL^−1^	Reducing Sugars mg g^−1^ DW
***In vitro***					
**0.0**	24.225 b	7.665 bc	31.891 c	6.610 b	63.623 d
**10**	26.232 b	9.395 b	35.627 b	7.751 a	132.10 c
**20**	33.795 a	11.267 a	45.062 a	8.591 a	195.30 b
**30**	20.989 c	6.354 c	27.343 d	5.740 b	260.059 a
**LSD_0.05_**	**1.728**	**1.730**	**2.881**	**1.007**	**2.234**
***Ex vitro***					
**0.0**	27.283 c	10.82 a	38.099 c	7.79 a	24.962 d
**10**	30.993 b	11.12 a	42.113 b	8.019 c	55.157 c
**20**	34.278 a	11.60 a	45.895 a	8.454 a	142.359 b
**30**	24.514 d	8.898 b	33.412 d	6.030 b	304.604 a
**LSD_0.05_**	**1.621**	**1.243**	**2.117**	**1.514**	**2.823**

Means followed by different letters are significantly different at *p* ≤ 0.05 according to LSD test.

**Table 5:** Effect of PEG *in vitro* hardening application on survival percentage (%) of date palm plants at the end of the 4th week from transferring to the greenhouse.

Treatments	Survival Percentage (%)
PEG 0.0 g L^−1^	63% ab
PEG 10 g L^−1^	72% ab
PEG 20 g L^−1^	90% a
PEG 30 g L^−1^	45% b
LSD_0.05_	27.73

Means followed by different letters are significantly different at *p* ≤ 0.05 according to LSD test.

## References

[1] Al-Khayri J., Jain S., Haggman H. (2007). Date palm *Phoenix dactylifera* L. micropropagation. Protocols for Micropropagation of Woody Trees and Fruits.

[2] Abd El Bar O.H.A., El Dawayati M.M. (2014). Histological changes on regeneration *in vitro* culture of date palm (*Phoenix dactylifera*) leaf explants. Aust. J. Crop Sci..

[3] Al-Khayri J.M. (2003). *In vitro* germination of somatic embryos in date palm: Effect of auxin concentration and strength of MS salts. Curr. Sci..

[4] Quiroz-Figueroa F.R., Rojas-Herrera R., Galaz-Avalos R.M., Loyola-Vargas V.M. (2006). Embryo production through somatic embryogenesis can be used to study cell differentiation in plants. Plant Cell Tissue Organ Cult.

[5] Al Kaabi H., Rhiss A., Hassan M. Effect of auxins and cytokinins on the *in vitro* production of date palm bud generative tissues and on the number of differentiated buds. Proceedings of the Second International Conference on Date Palms.

[6] Al-Khateeb A. (2006). Role of cytokinin and auxin on the multiplication stage of date palm (*Phoenix dactylifera* L.) cv. Sukry. Biotechnology.

[7] Hazarika B., Parthasarathy V., Nagaraju V. (2004). Influence of *in vitro* preconditioning of *Citrus* sp. microshoots with sucrose on their *ex vitro* establishment. Ind. J. Hortic..

[8] Pospóšilová J., Tichá I., Kadleček P., Haisel D., Plzáková Š. (1999). Acclimatization of micropropagated plants to *ex vitro* conditions. Biol. Plant..

[9] Pospisilova J., Synková H., Haisel D., Semoradova S. (2007). Acclimation of plantlets to *ex vitro* conditions: Effects of air humidity, irradiance, CO_2_ concentration and abscisic acid (a Review). Acta Hortic..

[10] Hazarika B. (2006). Morpho-physiological disorders in *in vitro* culture of plants. Sci. Hortic..

[11] Chandra S., Bandopadhyay R., Kumar V., Chandra R. (2010). Acclimatization of tissue cultured plantlets: From laboratory to land. Biotech. Lett..

[12] Fabbri A., Sutter E., Dunston S.K. (1986). Anatomical changes in persistent leaves of tissuecultured strawberry plants after removal from culture. Sci. Hortic..

[13] Asayesh Z.M., Vahdati K., Aliniaeifard S., Askari N. (2017). Enhancement of *ex vitro* acclimation of walnut plantlets through modification of stomatal characteristics *in vitro*. Sci. Hortic..

[14] Vahdati K., Asayesh Z.M., Aliniaeifard S., Leslie C. (2017). Improvement of *ex vitro* desiccation through elevation of CO_2_ concentration in the atmosphere of culture vessels during *in vitro* growth. HortScience.

[15] Awad M.A. (2008). Promotive effects of a 5-aminolevulinic acid-based fertilizer on growth of tissue culture-derived date palm plants (*Phoenix dactylifera* L.) during acclimatization. Sci. Hortic..

[16] Awad M.A., Soaud A., El-Konaissi S. (2006). Effect of exogenous application of anti-stress substances and elemental sulfur on growth and stress tolerance of tissue culture derived plantlets of date palm (*Phoenix dactylifera* L.) cv ‘Khalas’ during acclimatization. J. Appl. Hortic..

[17] Zaid A., de Wet P.F., Zaid A., Arias-Jimenez E. (1999). Date palm propagation. Date Palm Cultivation.

[18] Pospíšilová J., Wilhelmová N.A., Synková H., Čatský J., Krebs D., Tichá I., Hanáčková B., Snopek J. (1998). Acclimation of tobacco plantlets to *ex vitro* conditions as affected by application of abscisic acid. J. Exp. Bot..

[19] Sutter E.G. (1985). Morphological, physical and chemical characteristics of epicuticular wax on ornamental plants regenerated *in vitro*. Ann. Bot..

[20] Zaid A., Hughes H. (1995). *In vitro* acclimatization of date palm (*Phoenix dactylifera* L.) plantlets: A quantitative comparison of epicuticular leaf wax as a function of polyethylene glycol treatment. Plant Cell Rep..

[21] Asayesh Z.M., Vahdati K., Aliniaeifard S., Askari N. (2017). Investigation of physiological components involved in low water conservation capacity of *in vitro* walnut plants. Sci. Hortic..

[22] Dias M., Pinto G., Santos C. (2011). Acclimatization of micropropagated plantlets induces an antioxidative burst: A case study with *Ulmus minor* Mill. Photosynth.

[23] Pospíšilová J., Synková H., Haisel D., Baťková P. (2009). Effect of abscisic acid on photosynthetic parameters during *ex vitro* transfer of micropropagated tobacco plantlets. Bio. Plant..

[24] Dami I., Hughes H. (1995). Leaf anatomy and water loss of *in vitro* PEG-treated ‘Valiant’ grape. Plant Cell Tissue Organ Cult..

[25] Zaid A., Hughes H. (1995). Water loss and polyethylene glycol-mediated acclimatization of *in vitro*-grown seedlings of 5 cultivars of date palm (*Phoenix dactylifera* L.) plantlets. Plant Cell Rep..

[26] Short K., Warburton J., Roberts A. (1987). *In vitro* hardening of cultured cauliflower and chrysanthemum plantlets to humidity. Acta Hortic..

[27] El Dawayati M.M., Bar O.H.A.E., Zaid Z.E., El Din A.F.Z. (2012). *In vitro* morpho-histological studies of newly developed embryos from abnormal malformed embryos of date palm cv. Gundila under desiccation effect of polyethelyne glycol treatments. Ann. Agric. Sci..

[28] Ali-Ahmad M., Hughes H.G., Safadi F. (1998). Studies on stomatal function, epicuticular wax and stem-root transition region of polyethylene glycol-treated and nontreated *in vitro* grape plantlets. Vitr. Cell. Dev. Biol. Plant.

[29] Aliniaeifard S., Asayesh Z.M., Driver J., Vahdati K. (2020). Stomatal features and desiccation responses of Persian walnut leaf as caused by *in vitro* stimuli aimed at stomatal closure. Trees.

[30] Hazarika B., Nagaraju V., Parthasarathy V., Bhowmik G. (2001). Biochemical basis of acclimatization of micropropagated plantlets–A review. Agric. Rev..

[31] Taiz L., Zeiger E. (2006). Plant Physiology: Stress Physiology.

[32] Evert R.F. (2006). Esau’s Plant Anatomy: Meristems, Cells, and Tissues of the Plant Body: Their Structure, Function, and Development.

[33] Joshi P., Joshi N., Purohit S. (2006). Stomatal characteristics during micropropagation of Wrightia tomentosa. Biol. Plan..

[34] Wardle K., Dobbs E.B., Short K.C. (1983). *In vitro* acclimatization of aseptically cultured plantets to humidity. J. Am. Soc. Hortic. Sci..

[35] Brainerd K., Fuchigami L. (1982). Stomatal functioning of *in vitro* and greenhouse apple leaves in darkness, mannitol, ABA, and CO_2_. J. Exp. Bot..

[36] Sutter E. (1988). Stomatal and cuticular water loss from apple, cherry, and sweetgum plants after removal from *in vitro* culture. J. Am. Soci. Hortic. Sci..

[37] Abd Elbar O.H., Farag R.E., Shehata S.A. (2019). Effect of putrescine application on some growth, biochemical and anatomical characteristics of *Thymus vulgaris* L. under drought stress. Ann. Agric. Sci..

[38] Guenni O., Douglas M., Baruch Z. (2002). Responses to drought of five Brachiaria species. I. Biomass production, leaf growth, root distribution, water use and forage quality. Plant Soil.

[39] Kim T.H., Bohmer M., Hu H.H., Nishimura N., Schroeder J.I. (2010). Guard Cell Signal Transduction Network: Advances in Understanding Abscisic Acid, CO_2_, and Ca^2^^+^ Signaling. Annu. Rev. Plant Biol..

[40] Arve L., Torre S., Olsen J., Tanino K. (2011). Stomatal responses to drought stress and air humidity. Abiotic Stress in Plants-Mechanisms and Adaptations.

[41] Riederer M., Schneider G. (1990). The effect of the environment on the permeability and composition of Citrus leaf cuticles. Planta.

[42] Kosma D.K., Jenks M.A., Jenks M.A., Hasegawa P.M., Jain S.M. (2007). Eco-physiological and molecular-genetic determinants of plant cuticle function in drought and salt stress tolerance. Advances in Molecular Breeding toward Drought and Salt Tolerant Crops.

[43] Bi H., Kovalchuk N., Langridge P., Tricker P.J., Lopato S., Borisjuk N. (2017). The impact of drought on wheat leaf cuticle properties. BMC Plant Biol..

[44] Safadi F., Hughes H. (1990). Comparison of the diffusive resistance of Polyethylene glycol treated and non-treated tissue culture Tobacco plantlets. HortScience.

[45] Elmaghrabi A.M., Rogers H.J., Francis D., Ochatt S.J. (2017). PEG induces high expression of the cell cycle checkpoint gene WEE1 in embryogenic callus of Medicago truncatula: Potential link between cell cycle checkpoint regulation and osmotic stress. Front. Plant Sci..

[46] Abd El-Maboud M.M.A., Abd Elbar O.H. (2020). Adaptive responses of *Limoniastrum monopetalum* (L.) Boiss. growing naturally at different habitats. Plant Physiol. Rep..

[47] Abd Elbar O.H. (2015). Development of the successive cambia in Sesuvium verrucosum Raf (Aizoaceae). Ann. Agric. Sci..

[48] Cha-um S., Takabe T., Kirdmanee C. (2012). Physio-biochemical responses of oil palm (*Elaeis guineensis* Jacq.) seedlings to mannitol-and polyethylene glycol-induced iso-osmotic stresses. Plant Prod. Sci..

[49] Zhang J., Jia W., Yang J., Ismail A.M. (2006). Role of ABA in integrating plant responses to drought and salt stresses. Field Crop. Res..

[50] Kong L., Yeung E.C. (1995). Effects of silver nitrate and polyethylene glycol on white spruce (*Picea glauca*) somatic embryo development: Enhancing cotyledonary embryo formation and endogenous ABA content. Physiol. Plant..

[51] Darko E., Végh B., Khalil R., Marček T., Szalai G., Pál M., Janda T. (2019). Metabolic responses of wheat seedlings to osmotic stress induced by various osmolytes under iso-osmotic conditions. PLoS ONE.

[52] Han B., Duan X., Wang Y., Zhu K., Zhang J., Wang R., Hu H., Qi F., Pan J., Yan Y. (2017). Methane protects against polyethylene glycol-induced osmotic stress in maize by improving sugar and ascorbic acid metabolism. Sci. Rep..

[53] Stasolla C., van Zyl L., Egertsdotter U., Craig D., Liu W., Sederoff R.R. (2003). The effects of polyethylene glycol on gene expression of developing white spruce somatic embryos. Plant Physiol..

[54] Handa S., Bressan R.A., Handa A.K., Carpita N.C., Hasegawa P.M. (1983). Solutes contributing to osmotic adjustment in cultured plant cells adapted to water stress. Plant Physiol..

[55] Zanella M., Borghi G.L., Pirone C., Thalmann M., Pazmino D., Costa A., Santelia D., Trost P., Sparla F. (2016). β-amylase 1 (BAM1) degrades transitory starch to sustain proline biosynthesis during drought stress. J. Exp. Bot..

[56] Wilson S.M., Burton R.A., Doblin M.S., Stone B.A., Newbigin E.J., Fincher G.B., Bacic A. (2006). Temporal and spatial appearance of wall polysaccharides during cellularization of barley (*Hordeum vulgare*) endosperm. Planta.

[57] Hazarika B. (2003). Acclimatization of tissue-cultured plants. Curr. Sci..

[58] Shim S.-W., Hahn E.-J., Paek K.-Y. (2003). *In vitro* and *ex vitro* growth of grapevine rootstock5BB’as influenced by number of air exchanges and the presence or absence of sucrose in culture media. Plant Cell Tissue Organ. Cult..

[59] Aliniaeifard S., Matamoros P.M., van Meeteren U. (2014). Stomatal malfunctioning under low VPD conditions: Induced by alterations in stomatal morphology and leaf anatomy or in the ABA signaling?. Physiol. Plant..

[60] Ziv M., Schwartz A., Fleminger D. (1987). Malfunctioning stomata in vitreous leaves of carnation (*Dianthus caryophyllus*) plants propagated *in vitro*; implications for hardening. Plant Sci..

[61] Murashige T., Skoog F. (1962). A revised medium for rapid growth and bio assays with tobacco tissue cultures. Physiol. Plant..

[62] Abdelbar O.H. (2017). Histological analysis of the developmental stages of direct somatic embryogenesis induced from *in vitro* leaf explants of date palm. Date Palm Biotechnology Protocols.

[63] Harley M., Ferguson I. (1990). The role of the SEM in pollen morphology and plant systematics. Scanning Electron Microscopy in Taxonomy and Functional Morphology.

[64] Moran R. (1982). Formulae for determination of chlorophyllous pigments extracted with N, N-dimethylformamide. Plant Physiol..

[65] Wellburn A.R. (1994). The spectral determination of chlorophylls a and b, as well as total carotenoids, using various solvents with spectrophotometers of different resolution. J. Plant Physiol..

[66] Marsden W.L., Gray P.P., Nippard G.J., Quinlan M.R. (1982). Evaluation of the DNS method for analysing lignocellulosic hydrolysates. J. Chem. Technol. Biotechnol..

[67] Snedecor G.W., Cochran W.G. (1980). Statistical Methods.

[68] Gomez K.A., Gomez A.A. (1984). Statistical Procedures for Agricultural Research.

